# Reply to the use of tissue characterization using cardiac magnetic resonance imaging and response to cardiac resynchronization therapy

**DOI:** 10.1093/europace/euaf101

**Published:** 2025-05-10

**Authors:** Alphonsus Liew, Sandra Howell, Christopher Aldo Rinaldi

**Affiliations:** Raynes Institute, King’s College London, SE1 7EH London, United Kingdom; Department of Cardiology, Guy’s and St Thomas’ NHS Foundation Trust, SE1 7EH London, United Kingdom; Raynes Institute, King’s College London, SE1 7EH London, United Kingdom; Department of Cardiology, Guy’s and St Thomas’ NHS Foundation Trust, SE1 7EH London, United Kingdom; Raynes Institute, King’s College London, SE1 7EH London, United Kingdom; Department of Cardiology, Guy’s and St Thomas’ NHS Foundation Trust, SE1 7EH London, United Kingdom

We read with interest the recently published manuscript in this journal by Kim *et al.* which explored the utility of tissue characterization by cardiac magnetic resonance (CMR) in predicting response to cardiac resynchronization therapy (CRT).^1^ We applaud Kim *et al.* for further clarifying the role of T1, T2, and extracellular volume (ECV) parameters in predicting positive CRT responders and reinforcing the role of CMR for patients undergoing CRT.

However, the location of scar as detected by late gadolinium enhancement (LGE) was not defined in this study, as acknowledged by Kim *et al.* This is pertinent because the negative clinical outcomes associated with scar, as demonstrated in previous studies, were only observed when the left ventricular (LV) lead was deployed in the region of scar.^2,3^ To this end, our group previously demonstrated real-time integration of CMR-detected scar and areas of dyssynchrony onto live fluoroscopy images to guide LV lead implantation away from the areas of scar and towards area of latest activation.^4^ This demonstrated acute success including shorter paced QRS duration and lower LV capture threshold compared with pacing in the areas of scar. Therefore, patients with an LGE burden of >20% and ECV of >34% who would be predicted to be non-responders based on the outcome of this study but do not have scar in the LV-paced region may in fact be CRT responders.

Furthermore, we question the timing of CRT-response measurement in this study which measured CRT response from 3 months after CRT implantation. While there is no universal definition of CRT response, this has conventionally been the improvement of left ventricular end systolic volume (LVESV) of ≥15% at 6 months post-CRT. In fact, yet unpublished results from our *post hoc* analysis of the MORE-CRT MPP trial^5^ demonstrated that a significant proportion of non-responders at 6 months were found to be responders at 12 months follow-up based on an improvement of LVESV ≥ 15%. In the same *post hoc* analysis, we found that patients with delayed response had similar clinical outcomes to early responders in heart failure hospitalization and death. This is in line the findings of the *post hoc* analysis of the REVERSE trial that found that the ‘stabilized’ group who had a 0 to <15% decrease in LVESV index at 6 months had similar rates of survival as the ‘improved’ group who had a decrease of ≥15% in LVESV index.^6^ In contrast, the ‘worsened’ group who had an increase in LVESV index, had far worse survival compared with the stabilized and improved groups. This highlights not only the importance of the timing of evaluation of CRT response but also the classification of CRT response into those who have improved, stabilized, and worsened. We encourage the authors to consider reclassifying their CRT response as described and to redefine CRT responders as those with LVESV improvement at least 6 months after CRT implantation to avoid underestimating the proportion of CRT responders in their study. The results of this study, while encouraging, should therefore be interpreted with caution.
